# Clickstream Data Yields High-Resolution Maps of Science

**DOI:** 10.1371/journal.pone.0004803

**Published:** 2009-03-11

**Authors:** Johan Bollen, Herbert Van de Sompel, Aric Hagberg, Luis Bettencourt, Ryan Chute, Marko A. Rodriguez, Lyudmila Balakireva

**Affiliations:** 1 Digital Library Research and Prototyping Team, Research Library, Los Alamos National Laboratory, Los Alamos, New Mexico, United States of America; 2 Theoretical Division, Mathematical Modeling and Analysis Group, and Center for Nonlinear Studies, Los Alamos National Laboratory, Los Alamos, New Mexico, United States of America; 3 Santa Fe Institute, Santa Fe, New Mexico, United States of America; Science Commons, United States of America

## Abstract

**Background:**

Intricate maps of science have been created from citation data to visualize the structure of scientific activity. However, most scientific publications are now accessed online. Scholarly web portals record detailed log data at a scale that exceeds the number of all existing citations combined. Such log data is recorded immediately upon publication and keeps track of the sequences of user requests (clickstreams) that are issued by a variety of users across many different domains. Given these advantages of log datasets over citation data, we investigate whether they can produce high-resolution, more current maps of science.

**Methodology:**

Over the course of 2007 and 2008, we collected nearly 1 billion user interactions recorded by the scholarly web portals of some of the most significant publishers, aggregators and institutional consortia. The resulting reference data set covers a significant part of world-wide use of scholarly web portals in 2006, and provides a balanced coverage of the humanities, social sciences, and natural sciences. A journal clickstream model, i.e. a first-order Markov chain, was extracted from the sequences of user interactions in the logs. The clickstream model was validated by comparing it to the Getty Research Institute's Architecture and Art Thesaurus. The resulting model was visualized as a journal network that outlines the relationships between various scientific domains and clarifies the connection of the social sciences and humanities to the natural sciences.

**Conclusions:**

Maps of science resulting from large-scale clickstream data provide a detailed, contemporary view of scientific activity and correct the underrepresentation of the social sciences and humanities that is commonly found in citation data.

## Introduction

Maps of science derived from citation data [1], [2], [3], [4], [5], [6], [7] visualize the relationships among scholarly publications or disciplines. They are valuable instruments for exploring the structure and evolution of scholarly activity. Much like early world charts, these maps of science provide an overall visual perspective of science as well as a reference system that stimulates further exploration. However, these maps are also significantly biased due to the nature of the citation data from which they are derived: existing citation databases overrepresent the natural sciences; substantial delays typical of journal publication [8], [9], [10] yield insights in science past, not present; and connections between scientific disciplines are tracked in a manner that ignores informal cross-fertilization.

Scientific publications are now predominantly accessed online. Scholarly web portals provide access to publications in the natural sciences, social sciences and humanities. They routinely log the interactions of users with their collections. The resulting log datasets have a set of attractive characteristics when compared to citation datasets. First, the number of logged interactions now greatly surpasses the volume of all existing citations. This is illustrated by Elsevier's announcement, in 2006, of 1 billion (1×10^9^) article downloads since the launch of its Science Direct portal in April 1999. In contrast, around the time of Elsevier's announcement, the total number of citations in Thomson Scientific's Web of Science from the year 1900 to the present does not surpass 600 million (6×10^8^). Second, log datasets reflect the activities of a larger community as they record the interactions of all users of scholarly portals, including scientific authors, practitioners of science, and the informed public. In contrast, citation datasets only reflect the activities of scholarly authors. Third, log datasets reflect scholarly dynamics in real-time because web portals record user interactions as soon as an article becomes available at the time of its online publication [8], [9]. In contrast, a published article faces significant delays before it eventually appears in citation datasets: it first needs to be cited in a new article that itself faces publication delays [11], [12], and subsequently those citations need to be picked up by citation databases.

Given the aforementioned characteristics of scholarly log data, we investigated a methodological issue: can valid, high resolution maps of science be derived from clickstream data and can clickstream data be leveraged to yield meaningful insights in the structure and dynamics of scholarly behavior? To do this we first aggregated log datasets from a variety of scholarly web portals, created and analyzed a clickstream model of journal relationships from the aggregate log dataset, and finally visualized these journal relationships in a first-ever map of science derived from scholarly log data.

## Methods

### Data collection

We aggregated a log dataset that contains approximately 1 billion (1×10^9^) user interactions. These interactions were logged in the course of 2006 and 2007 by web portals operated by the following scientific publishers, aggregators, and institutions: Thomson Scientific (Web of Science), Elsevier (Scopus), JSTOR, Ingenta, University of Texas (9 campuses, 6 health institutions), and California State University (23 campuses). Strict confidentiality agreements prevent the distribution of any comparable and identifiable statistics with regards to individual web portals. However, the results of the analysis of aggregated log data across web portals, such as our map of science, can be freely published.

These distinct portals were selected for two reasons. First, their log data tracks user interactions across the boundaries of individual publisher collections. Second, the resulting aggregate log data set was expected to cover sources in the natural sciences, social sciences, as well as the humanities.

From this aggregate log dataset, we selected a subset that includes user interactions that occurred between March 1st 2006 and February 1st 2007 because this timeframe was covered by the logs of all aforementioned portals. The resulting log dataset contains 346,312,045 user interactions pertaining to 97,532 serial publications. Many of these publications are scholarly journals, but weekly magazines and newspapers such as The New York Times are also included.

We then processed this log dataset of individual interactions [13] to select only those that are considered expressions of interest by a user for a specific article, for example clicking links to request the full-text of the article or the abstract of the article. This process included removing interactions such as keyword searches and next page requests, as well as those that could straightforwardly be attributed to web crawlers by means of their hostnames. Finally, consecutive expressions of interest by a user in the same article in the course of the same session were interpreted as a single expression of interest in the article.

### Journal domain classification

In order to assign a general scientific discipline to each journal we extracted journal classifications from two databases, namely Thomson Scientific's Journal Citation Reports (JCR) classification codes (approx. 8,000 journals in the Natural Science and Social Science edition) and the Dewey Decimal system (approx. 40,000 journals) as provided by Ulrich's Serials Directory. These are the two most complete, prominent and widely applied journal subject classifications available.

JCR and Dewey Decimal classification codes were retrieved for each journal in our logs. However, the JCR and Dewey Decimal classification systems do not organize their classification codes into a common taxonomy, meaning that no comparison can be made between JCR and Dewey Decimal classifications at various levels of abstraction, e.g. JCR's “medicine” vs. Dewey Decimal's “Medical sciences – Oncology”. The JCR and Dewey Classification codes were therefore manually mapped to the Disciplines hierarchy of the Getty Research Institute's Art and Architecture Thesaurus (AAT)[14] that was used as a unifying, taxonomic classification structure. This involved the mapping of 215 JCR classification codes into 202 matching AAT disciplines and 425 Ulrich Dewey Values into 98 matching AAT disciplines, at various depths in the AAT taxonomy.

**Figure 1:**
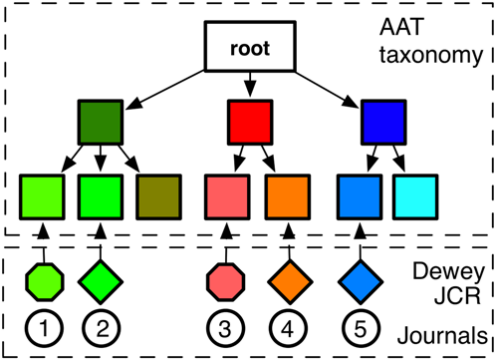
Matching JCR and Dewey Journal classifications to the AAT taxonomy.

The AAT Disciplines hierarchy is structured as a taxonomical tree that starts by differentiating between the social sciences, humanities, natural sciences and interdisciplinary sciences, and splits these broad categories into increasingly finer subject areas. As such we could place each journal in our log data at a branch in the AAT taxonomy as shown in Fig. 1. A JCR or Dewey Decimal classification code and matching AAT taxonomy position could be assigned to the journals involved in 93% of all interaction events.

The distribution of journal domain classifications for the log data obtained from each provider indicates its degree of coverage for the different domains in the AAT. The domain distribution obtained from pooling interaction events over all data providers, as shown in Table 1, reveals log data in which interaction events pertain to journals in the social sciences (47%) and natural sciences (41%) in nearly equal numbers. In addition, the humanities correspond to roughly 8% of all interaction events, while interdisciplinary fields account for 3%. This distribution deviates only slightly from the distribution of degrees conferred in the entire University of California (UC) system in 2007 by domain. Although it is not feasible to perform a full census of the scientific community, this indicates that the representation of scientific disciplines in our usage data, conforms at least to that observed in a large, diverse scientific community such as the UC system.

**Table 1:** Comparison of journal domain classifications in usage data set to JCR (Science and Social Science edition combined) and UC degrees conferred in 2006.

Domain	Usage	UC Degrees	JCR
Natural Science	37%	39%	92.8%
Social Sciences	45%	46%	7.2%
Humanities	14%	15%	

Source: http://www.ucop.edu/ucophome/uwnews/stat/statsum/fall2007/statsumm2007.pdf (table 9).

The discipline coverage of our log dataset can be contrasted to the coverage provided by the JCR, a citation database that is commonly used in the construction of journal-based science maps. When analyzing the total amount of citations in the Science vs. Social Science edition of the 2007 edition of JCR, a distribution of journal domains emerges that is heavily skewed towards the natural sciences as opposed to the social sciences and humanities, respectively 92.8% vs. 7.2%.

### A clickstream model of journal relationships

For each user interaction the resulting dataset contained the following data elements:

#### Article identifier

Or sufficient metadata to identify the article to which the interaction pertained.

#### Date-time

A date-time of the interaction, to the second.

#### Session identifier

A session identifier assigned by the web portal at the start of a user's information gathering session [15].

**Figure 2:**
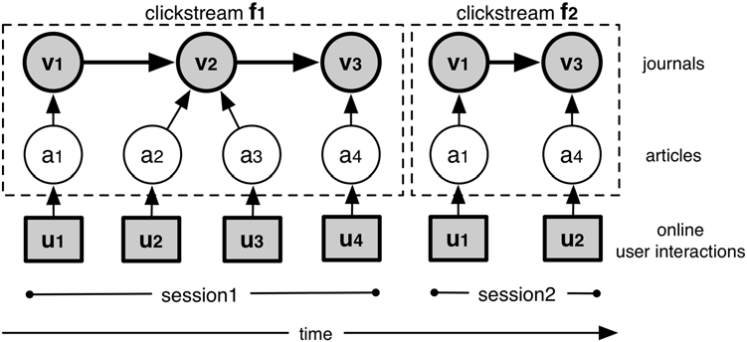
The extraction of journal clickstream data from article level log data. Usage log data consists of sequences of timed interaction events 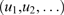. Interaction events issued by the same user from the same client can be grouped in user sessions. Each user session represents a clickstream 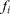 that can be expressed as a sequence of the articles that were part of the session's interaction events, i.e. 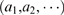. Since every article is published in a journal, we can derive journal clickstreams, i.e. 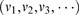. From the collection of all journal clickstreams we can calculate the probability 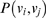.

We use the session identifier and date-time to reconstruct temporal sequences [16], [17] of interactions by the same user. These sequences can be mapped to article clickstreams, each of which records the navigation of a user from one article to another [18], [19]. Since each article is published in a journal, these article clickstreams can be translated to journal clickstreams. The resulting data set is a collection of journal clickstreams that reflects the navigation of users from one journal to another when interacting with scholarly web portals (Fig. 2).

We used this dataset to compute relationships among journals on the basis of their joint occurrences in the resulting journal clickstreams. We did so by using a method similar to association rule learning [20] that is commonly used in data mining applications and that is based on the co-occurrence principle. Applied to our case, this principle states that a journal 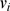 is related to a journal 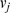 if 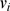 directly precedes 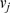 within a journal clickstream; the strength of the relationship between 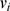 and 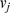 is expressed as the probability by which one follows the other over all journal clickstreams. When computing these journal relationships for the entire dataset, we effectively construct a stochastic model of how users move between pairs of journals in their online interactions.

More formally, we build a first-order Markov chain model of the clickstream data [21] in the following way. We define each recorded interaction 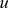 as a set that contains a session identifier 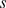, a date-time 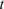 and the article 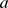 to which the interaction pertained, i.e. 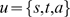. Our usage data log 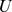 then consists of a set of 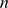 interactions 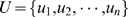. We now define 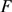 the set of clickstreams extracted from 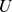, such that each element 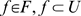 consists of an set of interactions with identical session identifiers, ordered by their data-time values, i.e. 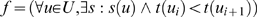 where 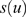 and 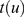 denote the session identifier and date-time of interaction 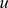 respectively.

Every interaction in the clickstream of 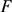 pertains to a particular article 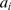. We can thus convert each 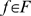 to an article clickstream 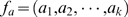. Likewise, since each article 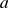 is published in a journal 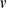, we can convert every article clickstream 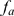 to journal clickstream 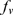 so that each 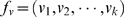.

Over all journal clickstreams we count the number of times 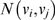 that a particular ordered journal pair 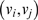 was observed. We do this for all pairs of journals 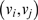 in which 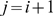, i.e. 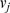 is immediately adjacent to 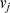 in the journal clickstream. Finally, we can calculate the transition probability
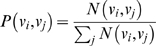
and form a matrix 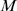 whose entries 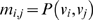.

Applying the described procedure to our log dataset results in a matrix 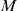 that has n = 97,532 rows and columns, corresponding to the number of unique journals, and contains 6,783,552 non-zero entries.

The journal relationships in 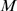 are intentionally directional, i.e. 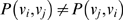, for two reasons. First, the entries of 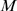 represent conditional probabilities derived from clickstream sequences, not symmetric journal similarities. The temporal order of user interactions induces a directed relation. Second, directed relations can be converted to undirected relations, but not vice versa. Maintaining the directionality of journal relations thus preserves information while at the same time establishing a foundation for additional analysis that may or may not rely on relation directionality.

### Visualization

**Figure 3:**
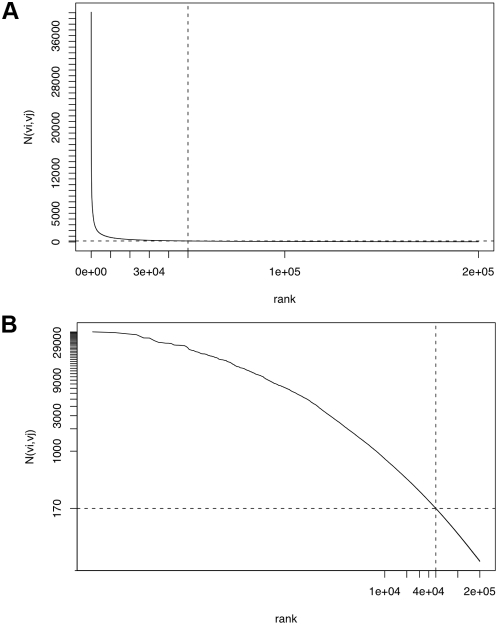
Distribution of edge weights in 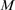.

To visualize a clickstream map of science on the basis of 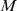 we proceeded as follows. To only use journal relationships for which we had a minimum number of observations to support the particular connection, we selected the 50,000 journal pairs with the highest 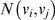 values. Although this threshold is arbitrary it corresponds to 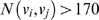, i.e. for each journal relationship we required at least 170 observations. The distributions in Fig. 3 show how this threshold approximates the distribution's “scree point”; it captures a wide range of edge weights while excluding journal relationships with relatively low 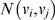 values.

**Figure 4:**
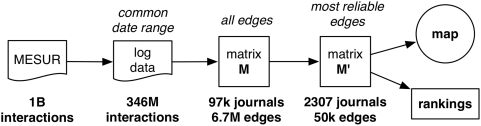
Summary of data processing leading to the map of science.

This set of journal relations pertained to 2,307 journals, and formed a reduced matrix 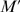. Table 2 list the network parameters of 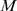 and 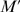, including matrix density. Fig. 4 provides a summary of the consecutive data processing steps that led to 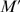.

**Table 2:** Network parameters of original (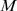) and reduced (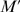) clickstream matrices.

Network matrix		
Parameter	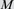	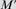
Journals	97,532	2,307
Edges	6,783,552	50,000
Matrix density	0.071%	0.939%
Strongly Connected Components (SCC)	16,474	236
Journals in SCC	80,934	1,944
Average journal clustering coefficient (SCC)	0.285	0.514
Diameter of largest SCC	37	14

To unclutter the map and show only the most relevant relationships per journal, we only retained the 5 strongest outbound relationships for each journal. Subsequently, we created a symmetric matrix 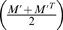 to obtain only a single edge for any journal pair in the visualization. From this matrix we selected the largest connected component to obtain a fully interconnected visualization.

Journals were then positioned in a map using the Fruchterman-Reingold (FR) network layout method [22], which optimizes journal positions so that they balance geometric node repulsion with node attraction resulting from the relationship strengths in 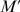. The distances between any pair of journals in the map correspond to the FR layout algorithm balancing these two forces on the basis of the entries of 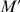. In the resulting map each circle represents a journal, connected to other journals. These connections are given by 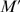. The radius 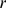 of each circle is scaled to the natural logarithm of the journal's degree centrality [23] 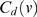, i.e. 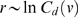, which is an indicator of the total amount of occurrences of the journal in 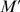, thus its importance to the visualization. The natural logarithm compresses the upper range of circle radius values to unclutter the map.

Color codes were assigned to each journal on the basis of its AAT discipline classification [14]. Colors were selected to achieve a maximal overlap with the color scheme proposed by Boyack and Klavans [24], according to which pink and blue indicate physics and chemistry, green indicates biology, red indicates medicine, and yellow and white represent social sciences and humanities, respectively. For the sake of clarity, individual journal titles were omitted. Instead, groups of journals are labeled according to the coarse-grained disciplines they cover.

**Figure 5:**
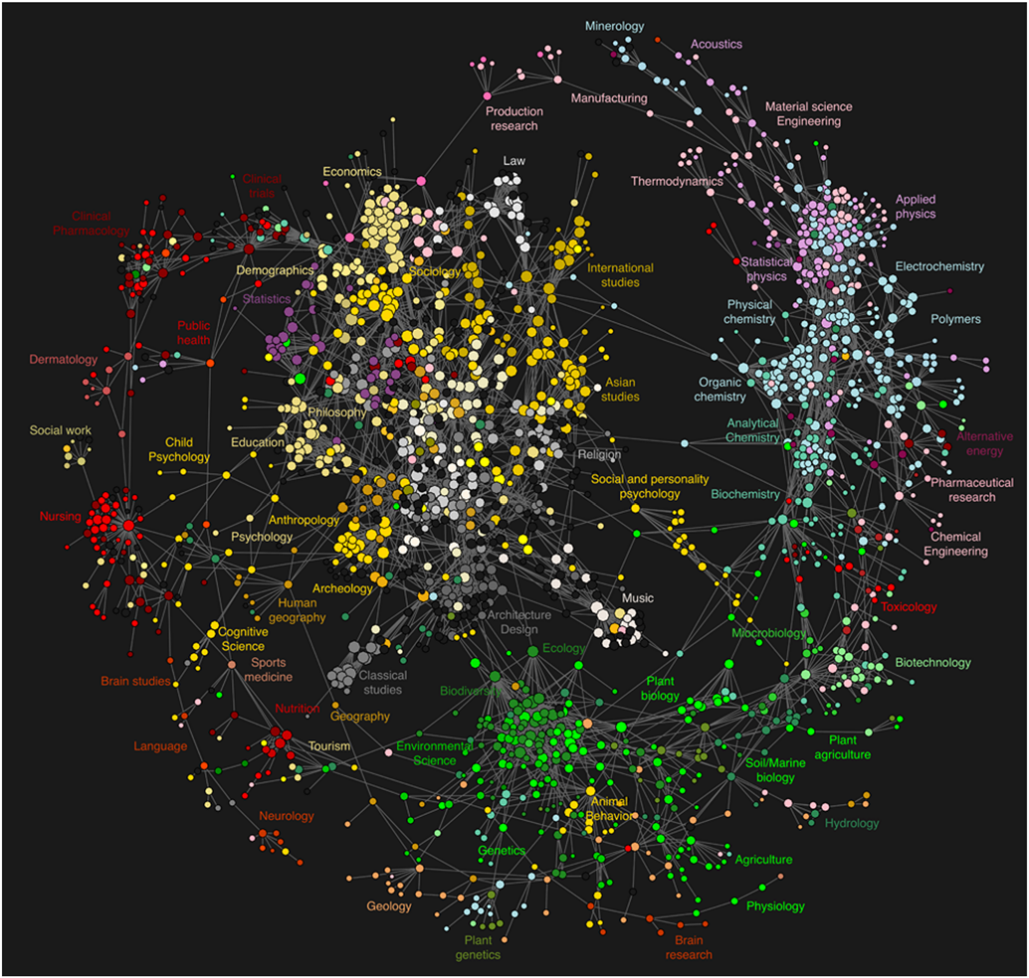
Map of science derived from clickstream data. Circles represent individual journals. The lines that connect journals are the edges of the clickstream model in 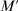. Colors correspond to the AAT classification of the journal. Labels have been assigned to local clusters of journals that correspond to particular scientific disciplines.

The resulting map is show in Fig. 5 and further discussed in the following sections.

## Results and Discussion

According to the above mentioned methodology we constructed a map of science that visualizes the relationships between journals according to user clickstreams. We first discuss the visual structure of the map, and then attempt to validate the structural features of its underlying clickstream model by comparing the latter to journal centrality rankings and an alternative model of journal relations derived from classification data.

### A clickstream map of science

Any interpretation of the visual structure of the map in Fig. 5 will be governed by the following considerations:

#### Convergence

The FR algorithm can converge on different visualizations of the same network data. We do not claim Fig. 5 is the only or best possible visualization. It was selected because it represents a particularly clear and uncluttered visualization of the connections between journals in 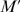, and most importantly, its main structural features were stable across many different iterations of the FR algorithm.

#### Connections

The journal connections shown in the map are given by 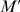, not the FR algorithm. They are thus not artifacts of the visualization.

#### Clustering

The FR algorithm will pull together small-scale clusters of journals that are strongly connected in 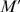. The appearance of small-scale journal clusters is thus directly related to the entries of 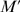 and they are thus not considered artifacts of the visualization.

#### Geometry

Although the positions of journals and clusters relative to each other are shaped by their connections in 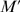, their exact geometric coordinates vary depending on the layout algorithm and are thus indeed considered artifacts of the visualization.

In summary, the connections between journals and small-scale clusters in the network visualization in Fig. 5 are determined by 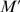. They are not artifacts of the visualization. However, one can not draw conclusions from the exact, geometrical coordinates of journals and clusters in the map.

To provide a visual frame of reference, we summarize the overall visual appearance of the map of science in Fig. 5 in terms of a wheel metaphor. The wheel's hub consists of a large inner cluster of tightly connected social sciences and humanities journals (white, yellow and gray). Domain classifications for the journals in this cluster include international studies, Asian studies, religion, music, architecture and design, classical studies, archeology, psychology, anthropology, education, philosophy, statistics, sociology, economics, and finance. The wheel's outer rim results from a myriad of connections in 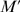 between journals in the natural sciences (red, green, blue). In clockwise order, starting at 1PM, the rim contains physics, chemistry, biology, brain research, health care and clinical trials journals. Finally, the wheel's spokes are given by connections in 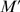 that point from journals in the central hub to the outer rim.

The connections between the journals in the map's rim cross multiple domains. For example, alternative energy (rim, 3PM) connects to pharmaceutical research and chemical engineering, which itself further connects to toxicology studies and biotechnology. Brain research (rim, 6PM) is connected to genetics, biology, animal behavior, and social and personality psychology. Human geography studies connects to geography, plant genetics, and finally agriculture. A number of clusters are well-connected to both the natural science and social science clusters. For example, ecology and biodiversity (5PM) connects the domains of biology (rim, 5PM) and architecture and design (hub, 5PM). Production and manufacturing (12PM) bridge the domains of physics and engineering (rim, 2PM) and economics (hub, 11PM).

### Validating the generated clickstream model

#### Journal centrality rankings

The map displays a dense, centrally located cluster of social science and humanities journals (hub). The question arises whether the central position of the social sciences and humanities journals is merely an artifact of the visualization, or whether these journals are in fact also central to the network topology of 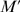.

To verify this, we calculated the betweenness centrality [25] (Table 3) and PageRank [26], [27] (Table 4) of all journals in 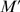. Each ranking highlights a different interpretation of a particular journal's centrality in 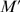.

**Table 3:** Ranking of journals from 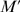 according to betweenness centrality.

Rank	Journal	Top-level AAT classification
1	Science	Natural Sciences
2	Proceedings of the National Academy of Sciences	Natural Sciences
3	Environmental Health Perspectives	Natural Science
4	Chemosphere	Natural Sciences
5	Journal of Advanced Nursing	Natural Sciences
6	Nature	Natural Sciences
7	Ecology	Natural Sciences
8	Milbank Quarterly	Natural Sciences
9	Applied and Environmental Microbiology	Natural Sciences
10	Child Development	Social Sciences
11	Behavioral Ecology and Sociobiology	Social Sciences
12	Journal of Colloid and Information Science	Natural Sciences
13	American Anthropologist	Social Sciences
14	Journal of Biogeography	Natural Sciences
15	Materials Science and Technology	Natural Sciences

**Table 4:** Ranking of journals from 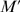 according to PageRank (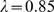).

Rank	Journal	Top-level AAT classification
1	Applied Physics Letters	Natural Sciences
2	Journal of Advanced Nursing	Natural Sciences
3	Journal of the American Chemical Society	Natural Sciences
4	Ecology	Natural Sciences
5	Nature	Natural Sciences
6	Physical Review B	Natural Sciences
7	Journal of Applied Physics	Natural Sciences
8	American Economic Review	Social Sciences
9	American Historical Review	Social Sciences
10	Physical Review Letters	Natural Sciences
11	Science	Natural Sciences
12	Langmuir	Natural Sciences
13	Journal of Chemical Physics	Natural Sciences
14	American Anthropologist	Social Sciences
15	Annals of the American Academy of Political and Social Science	Social Science

The betweenness centrality of a journal 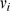 is defined as the number of geodesics (shortest paths) in 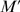 that pass through 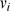. Let 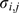 be the number of weighted shortest paths between journals 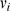 and 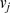 in the graph and 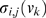 be the number of those shortest paths that pass through node 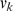. The weighted betweenness centrality of node 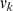 is then given by Equation 1:
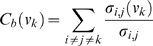
(1)


Journals with high betweenness centrality values are those that frequently sit on paths that connect a large number of other journals and journal clusters; they will often be interdisciplinary journals that serve as connectors between various domains. Table 3 lists the 15 journals with highest betweenness centrality; most of these journals are indeed highly inter-disciplinary such as Nature, Science, PNAS, Milbank Quarterly, Behavioral Ecology and Sociobiology. The presence of social science journals, such as Child Development and American Anthropologist, in this ranking confirms their interdisciplinary natures and overlaps with their central position in the map.

The PageRank of a journal is calculated by an iterative procedure in which the PageRank of a journal is continuously recalculated as a function of the PageRank of its predecessors in the graph, according to Equation 2.
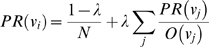
(2)where 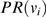 denotes the PageRank of journal 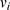, 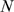 the number of nodes in 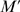, and 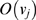 the out-degree of the predecessor journal 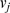. PageRank values converge from a set of random initial values toward a stable ranking after a given number of iterations.

PageRank favors prestigious journals that are well-connected to other well-connected journals. Table 3 list the 15 journals which the highest PageRank values in 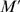; this ranking indeed favors more specialized, prestigious journals, such as Applied Physics Letters, Ecology, Physical Review B and American Anthropologist. The presence of social science and humanities journals in the PageRank ranking, such as American Historical review and Annals of the American Academy of Political and Social Science, indicates their connectedness to other highly ranking journals and subsequently their centrality in 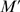.

Regardless of their use for cross-validating features of the produced map of science, the rankings in Table 3 and Table 4 illustrate the possibility of ranking journals according to various aspects of their centrality in clickstream data. For example, we note that Nature and Science are among the 15 top-ranked journals in both Table 3 and Table 4. This indicates that they have considerable interdisciplinary appeal as well as high prestige among users. The betweenness centrality and PageRank of PNAS diverge more strongly; PNAS was ranked 2nd in the betweenness centrality ranking, but 24th according to its PageRank. This suggests that PNAS has strong interdisciplinary appeal among users, but a slightly smaller degree of prestige compared to other top 15 journals.

### Cross-validation of the clickstream model and map to the AAT

The clickstream model represented by matrix 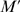 expresses the relations between pairs of journals. An inspection of the individual journal relationships in Table 5 may provide an informal sense of the validity of journal relations in 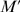. We selected 6 prominent journals, i.e. those with high 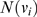 values, and retrieved the 5 journals with which they have the highest highest probability 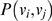 connection. All journal relations in Table 5 seem highly valid, but this is a subjective observation.

**Table 5:** Sample of journals pairs with high 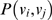.

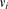	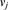	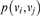	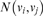	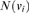
American Journal of International Law	International Organization	0.0207	9,292	448,034
	International Affairs	0.0184	8,254	
	International and Comparative Law Quarterly	0.0171	7,654	
	Foreign Policy	0.0167	7,500	
	American Political Science Association	0.0140	6,291	
Journal of Educational Sociology	American Journal of Sociology	0.0334	2,790	83,419
	Journal of Higher Education	0.0303	2,529	
	Journal of Negro Education	0.0286	2,389	
	American Sociological Review	0.0276	2,303	
	Social Forces	0.0249	2,076	
Surface Science	Physical Review B	0.0704	2,555	36,282
	Applied Surface Science	0.0341	1,239	
	Physical Review Letters	0.0339	1,230	
	Journal of Chemical Physics	0.0333	1,207	
	Applied Physics Letters	0.0327	1,188	
Journal of Organic Chemistry	Journal of the American Chemical Society	0.0873	4,141	47,439
	Tetrahedron Letters	0.0865	4,105	
	Tetrahedron	0.0602	2,857	
	Organic Letters	0.0532	2,526	
	Angewandte Chemie	0.0305	1,448	
Ecological Applications	Ecology	0.0965	13,659	141,481
	Conservation Biology	0.0524	7,408	
	Bioscience	0.0215	3,043	
	Annual Review of Ecology and Systematics	0.0215	3,043	
	Clinical and Experimental Allergy	0.0191	2,699	
Annals of Mathematics	American Journal of Mathematics	0.0705	5,392	76,526
	American Mathematical Monthly	0.0579	4,432	
	PNAS	0.0156	1,195	
	Econometrica	0.0082	624	
	Mathematics Magazine	0.0077	587	

However, we can cross-validate the map's structure, represented by matrix 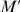, in a more objective manner by comparing it to an independent set of journal relations as demonstrated by [28]. Assume we create an alternative matrix of journal relations 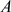 from an independent, yet trusted data source unrelated to our usage data. If 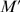
's entries correspond to the structure of 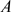, that finding corroborates the validity of the structure of matrix 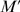.

To perform such cross-validation two conditions need to be satisfied:

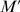
 and 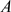 must be derived from independent data sources.

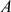
 needs to represent journal relations at various levels of granularity, above that of individual journal relations.

The AAT classification matches these requirements. First, the journal classifications in the AAT are derived from two well-established, commonly used classification schemes, namely Dewey Decimal and JCR classification codes. These were defined independent of our usage data and thus the relationships in 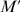. Second, the AAT expresses the classification of journals at various levels of granularity to which the structural features of our map can be compared.

We derived a model of journal relations, represented by matrix 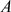, from the AAT as follows. We denote the AAT classification of journal 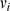 as 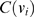. Since journal classifications can be retrieved from the AAT at various distances 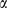 from the root of the taxonomy, we denote the journal classification of journal 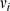 at root distance 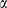 as 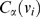.

For each journal pair 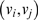 we can retrieve the corresponding AAT classification pair 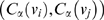. We thus define the match function 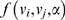 such that
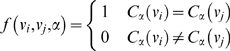


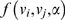
 maps each journal pair 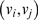 in 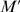 to a binary value depending on whether their AAT classifications match at the particular root distance 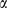.

We then define the AAT classification match matrix 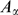 whose entries 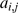 are given by 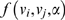; they represent a binary indication of journal relationships according to their AAT classifications. We can generate 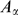 matrices at any root distance 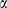. However, not all branches of the AAT taxonomy are equally represented at 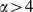. We therefore chose 4 values that provided a consistent range of classification granularities, namely 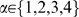 each of which corresponds to an increasingly detailed classification level with 4 being the most specific. The root distances 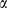 and the number of distinct classifications at that level in the taxonomy 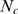 are listed in Table 6.

**Table 6:** Distance from AAT root (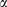) and number of classifications 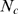 at that level. Each 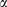 produces a finer-grained separation of scientific disciplines.

Distance ( 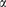 )	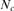	Example classifications
1	4	Natural sciences, social sciences, humanities, and interdisciplinary sciences, …
2	8	Biology, chemistry, physics, …
3	31	Classics, communication, engineering, …
4	195	Allergy, anesthesiology, applied linguistics, …

We now formulate the null-hypothesis 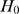 as follows:


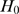
 = “Over all non-zero entries of 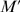, the magnitude of 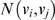 is not related to the probability that 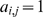.”

The probability of rejecting 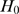 increases as 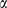 decreases, since classifications are being retrieved closer to the AAT root and thus result in increasingly general associations.

We test the stated null-hypothesis by performing a Pearson's 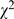 analysis (with Yates' continuity correction) on four 2×2 contingency tables constructed over a pairwise comparison of the non-zero entries of 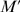 and 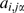 at each 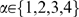.

For each non-zero entry in 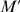 we thus compare the following two factors for the corresponding journal pair 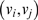:

**Factor 1** 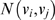 is either above or below the median of 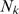 values, denoted 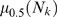


**vs.**

**Factor 2** 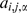 is either 0 or 1

where 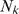 denotes the the set of all non-zero entries in 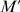.

If the set of journal connections in 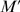 are unrelated to those given by their AAT classifications, i.e. if 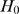 holds, we expect the frequencies in the cells of the 2×2 contingency tables to match those predicted from their sum- and row-totals on the assumption of statistical independence.

However, 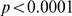 values were found at all 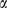 levels, i.e. for 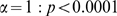, 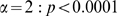, 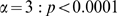, and 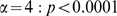. We can thus reject the null-hypothesis 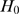 at high levels of confidence for each 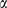 level, and conclude that the entries of 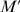 are indeed related to the AAT classifications of the journals 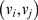 thereby corroborating the validity of 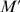 at least to the degree that the AAT can be considered a valid taxonomy.

**Figure 6:**
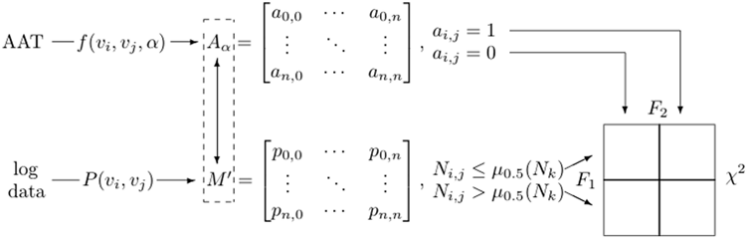
Cross-validating the map structure given by 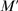 to journal relationships derived from AAT journal classifications, i.e. matrix 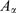.

Fig. 6 provides summary of the above mentioned procedure.

At 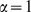 level the AAT distinguishes between 4 classifications: natural sciences, social sciences, humanities and interdisciplinary science. The null-hypothesis 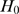 was rejected at this level indicating a statistically significant relation between journal relations in 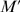 and the AAT classifications of the corresponding journals. To visually illustrate the overlap between journal relations in 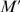 and their AAT classifications at 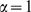, we assigned each journal a color according to its 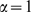 classification. The natural sciences were assigned the color blue, while the social sciences and humanities combined were assigned the color yellow. Since only a small fraction of journals (3%) were classified as inter-disciplinary they were colored gray along with all other journals that could not be classified.

**Figure 7:**
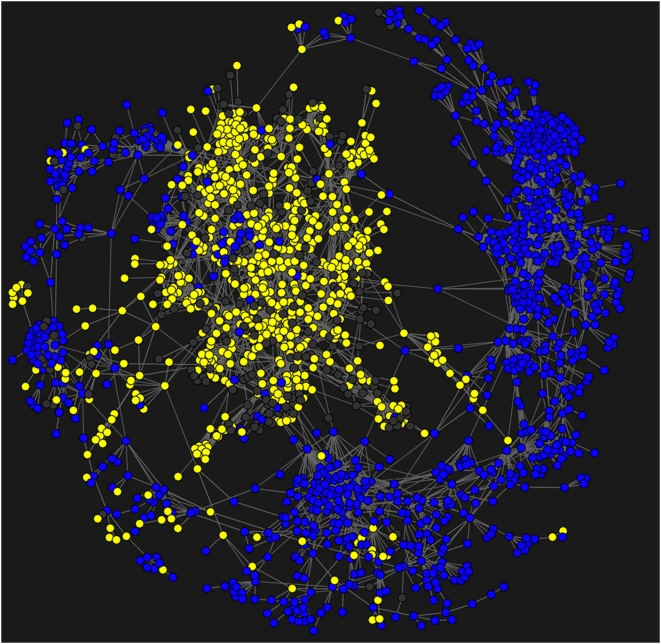
Cross-validating the map of science's layout by retrieving each journal's top-level AAT classification (natural sciences vs. social sciences and humanities). This map colors journals according to whether the AAT classifies them as either social sciences and humanities journals (yellow) vs. natural science journals (blue). Highly connected clusters corresponding to biology and psychology contain a mix of journals classified in either the social and natural sciences.

Fig. 7 results from this procedure; it shows the overlap between the AAT subject classifications and the map's layout of journals in the mentioned hub, rim and spokes, confirming that the visual separation of these domain effectively follows their separation according to the AAT subject classification.

The map shown in Fig. 7 also shows blue circles connected to journal in the central yellow hub, and yellow circles connected to journals in the blue rim. These discrepancies indicate a divergence between the AAT classification scheme compiled by *experts* vs. how journals are connected in the map according to 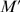, i.e. *user* clickstreams. For example, the AAT assigns numerous journals in biology, neurology and hydrology to the social sciences and humanities whereas their connections in 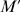 place them within the cluster of natural sciences (rim, 6PM). Conversely, several journals in clinical pharmacology and statistics are assigned to the natural sciences by the AAT although their connections place them within the cluster of social science and humanities journals (hub, 10PM). Psychology (rim, hub 8PM) is an example of a domain whose connections place it on the intersection of the social sciences and natural sciences. Psychology journals are nearly equally classified within both the natural sciences and the social sciences by the AAT.

### Future Research

This article seeks to address a basic methodological question: can accurate maps of science be derived from clickstream data? Our maps are the first of their kind and reveal numerous features of scientific activity. However, several pertinent issues require further study, but were outside the scope of this article.

First, users' clickstreams can be shaped by various navigation patterns. Users can follow citation links, follow the results of full-text searches, download articles on the basis of social recommendations, etc. Our clickstream map is thus necessarily the result of an overlay of an unknown combination of such navigation patterns. An analysis of the divergence between maps derived from usage, citation and text mining data might disambiguate the many influences that shape clickstream maps.

Second, when users navigate scholarly web portals their behavior will be shaped by the interfaces of the particular web portal [29]. In this paper we attempted to minimize such influences by aggregating usage log data from a variety of web portals. However, more research is necessary to determine the precise influence of interface effects on the creation of maps of science from clickstream data. In addition, the usefulness of various interaction types as indicators of user interest merits further investigation. For example, are full-text downloads stronger indicators of user interests than requests to view an article's abstract?

Third, we have adopted a lowest common denominator approach to building a clickstream model under first-order Markov Chain assumptions. Scholarly behavior may very well be more goal-oriented and less sporadic than web traffic. Our clickstream data lends itself well to tracing higher-order regularities in usage behavior. An investigation of models of usage behavior under various Markov assumptions and parameters will thus be an interesting venue for future research. Given our particular visualization methodology, i.e. network visualizations of pair-wise connected journals, it is however not certain that higher-order Markov models of our clickstream data will necessarily provide more accurate maps of science.

Finally, the promise of the deriving maps of science from usage log data lies in its ability to track scientific behavior as it takes place and track contemporary trends in scientific activity. Therefore future research will focus on a longitudinal, comparative analysis between citation maps and usage maps to determine the parameters of the relationship between usage and citation behavior. This however requires the collection, aggregation and analysis of additional usage and citation data which is forthcoming.

### Conclusion

Several web enterprises have successfully used clickstream data as a means to enhance their impact, for example through the introduction of recommender systems. Clickstream data of scholarly web portals have thus far not received significant attention. This is remarkable since the map of science that we described here, as well as other findings of our MESUR project [13], strongly suggest that scholarly logs hold valuable information about the dynamics of scholarship.

Log datasets have attractive characteristics when compared to citation datasets: they can be aggregated to cover all scholarly disciplines, and they reflect the activities of a broader scholarly community. But, most importantly, the immediacy of log datasets offers the possibility to study the dynamics of scholarship in real-time, not with a multi-year delay, as is currently the case with citation data. The resulting potential for a wide variety of analysis of the structure and dynamics of scholarship, such as trend analysis and prediction [30], should not be underestimated.

There can exist stark differences between what people claim they do and what they actually do [31]. This also applies to the distinction between citing behavior and online information seeking behavior. The first is a public and explicit expression of influence by scholarly authors, whereas the latter results from the private navigation behavior of scholarly users of web portals. This distinction leads to different insights regarding scholarly activity depending on whether it is mapped on the basis of citation data versus scholarly log data. Our map of science derived from clickstream data may thus run counter to accepted wisdom which is at this point mostly based on citation data, yet it offers a first-ever glimpse of this scholarly terra incognita.

Our map represents the structure of scholarly activity from an observational perspective, not from a prescriptive or motivational one. User interactions with scholarly web portals are shaped by many constraints, including citation links, search engine results, and user interface features. In this paper we do not attempt to explain or motivate these interactions, but merely to demonstrate how their overall structure can be charted and described from clickstream maps of science.

Maps constructed from clickstream data can serve numerous functions. Like citation maps they provide a means to visually assess the relationships between various domains and journals. However, clickstream maps of science can offer an immediate perspective on what is taking place in science and can thus aid the detection of emerging trends, inform funding agencies, and aid researchers in exploring the interdisciplinary relationships between various scientific disciplines. Clickstream maps can furthermore be used as the basis for exploration and recommendation services that rank journals according to the various parameters of network topology, so that researchers can identify influential journals in any particular domain of interest.

Scholarly log datasets still present some significant challenges. There is no established framework for the aggregation of datasets across web portals, there are no standards for recording logs, or for the determination of what exactly constitutes an expression of interest in a specific article. There are privacy concerns regarding users of web portals and concerns regarding the sharing of what ultimately is business intelligence by operators of web portals. And there is an understanding that clickstreams can be manipulated. As a matter of fact, the incentives to do so would increase if metrics for the assessment of impact of articles, journals, authors, departments and institutions derived from log data would become used as an addition to the established citation-based impact metrics. Determining the feasibility of such novel metrics is of significant importance to the scholarly community and has thus become the objective of several research initiatives including the MESUR project (http://www.mesur.org/).
